# An Improved GAS Algorithm

**DOI:** 10.3390/e27030240

**Published:** 2025-02-26

**Authors:** Zhijian Wang, Yuchen He, Tian Luan, Yong Long

**Affiliations:** 1 Department of Computer Technology, Institute of Advanced Technology, University of Science and Technology of China, No. 96 Jinzhai Road, Hefei 230088, China; wangzhijian@mail.ustc.edu.cn (Z.W.); longyong@mail.ustc.edu.cn (Y.L.); 2 Yangtze Delta Region Industrial Innovation Center of Quantum and Information Technology, Suzhou 215100, China; heyuchen@tgqs.net

**Keywords:** GAS, QAOA, quantum computing

## Abstract

This paper introduces an improved Grover Adaptive Search (GAS) algorithm. The GAS algorithm has been prove to achieve quadratic acceleration in the Constrained Polynomial Binary Optimization (CPBO) problem. Nevertheless, the acceleration effect of the GAS algorithm can be decreased by the poor threshold selection. This article uses the Quantum Approximate Optimization Algorithm (QAOA) to improve the initial threshold selection, thereby accelerating the convergence speed of the original GAS algorithm. The acceleration effect of the improved GAS algorithm is presented by the Max-Cut problem and the CPBO problem.

## 1. Introduction

Solving the large-scale quadratic unconstrained binary optimization (QUBO) [1,2] problem and CPBO problem with classical heuristic algorithms presents many challenges and disadvantages. In fact, the QUBO and CPBO problem is NP-hard [3,4], which means that as the size of the problem increases, exhaustively searching for all possible solutions to find the optimal one becomes infeasible due to the exponential growth of the complexity of time. Moreover, the classical heuristic algorithms (such as simulated annealing, genetic algorithms, and tabu search) are prone to becoming stuck in local optima. Consequently, these algorithms may not identify the optimal global solution for large or complex QUBO problems, frequently resulting in suboptimal solutions. Additionally, the objective function of the QUBO problem may consist of complex interaction terms, further increasing the difficulty of solving the problem. Although some classical algorithms can be parallelized to enhance performance, the efficiency of parallelization is frequently constrained by the intrinsic nature of the algorithm. In certain instances, parallelization may result in additional overhead and may not signally enhance the efficiency of the solution.

Compared with the classical algorithm, quantum algorithms show potential in solving the large-scale QUBO problem. The QAOA [5,6,7,8] leverages the properties of quantum mechanics to explore the solution space more efficiently. One potential benefit of quantum mechanics is that it can process multiple pieces of information at the same time. This ability can greatly improve the speed and efficiency of computation when dealing with large-scale problems. The GAS [9,10] algorithm is a quantum search algorithm based on the Grover [11,12] algorithm. It can be used to solve the CPBO [13] problem. The GAS algorithm utilizes an artificial threshold as the initial benchmark to identify all values that surpass it in the quest for a better solution. However, if the threshold is not appropriately chosen, the algorithm’s performance can degrade significantly, resulting in the loss of its acceleration advantage.

This paper introduces an improved GAS algorithm that uses the QAOA to select the initial threshold to avoid the problem of the algorithm’s performance degradation mentioned above when dealing with CPBO problems. We evaluate our algorithm both on a simplified credit card scoring problem (CPBO problem) and the Max-Cut [14] problem (QUBO problem). Comparing the experimental results, the average time complexity of the experiments with the improved GAS algorithm is 56.74% lower than the GAS method in the credit card scoring problem.

The remainder of this paper is organized as follows. In the Results section, we define the credit card scoring problem, describe the details of the improved GAS algorithm, and apply the proposed algorithm to both the credit card scoring problem and the Max-Cut problem. Finally, we provide a conclusion.

## 2. Define CPBO Problem

The credit card scoring problem is very important in the modern financial system. Usually, after designing an evaluation model, this problem can be converted to a CPBO problem to find the best credit cart portfolio. The Table 1 explains the meaning of the variable and objective function is given as

(1) $$F\left(x_{1},x_{2},\ldots ,x_{n}\right)=\sum_{\begin{array}{c} 0<i\leq n \end{array}}crt_{i}\left(1-h_{i}\right)x_{i}-ct_{i}h_{i}x_{i},\;x_{i}\in \left\{0,1\right\},\;n\in N,$$

where $x_{i}$ represents whether the credit card is picked, *n* is the total number of the credit cards, and $h_{i}\in \left[0,1\right]$, $c_{i}\in R$, $t_{i}\in \left[0,1\right]$ and r∈R represent the bad debt rate, the total loan, the loan approval rate, and the interest rate for card *i*, respectively.

In practice, several constraints have to be considered in the credit card scoring problem. Here, we consider a simple constraint $\sum_{\begin{array}{c} 0<i\leq n \end{array}}x_{i}=B.$

The CPBO problem is defined as

$$\underset{\begin{array}{c} x_{i}\in \left\{0,1\right\}\\ i\in \{1,2,\ldots ,n\} \end{array}}{\arg\max}F\left(x_{1},x_{2},\ldots ,x_{n}\right),\;s.t.\sum_{\begin{array}{c} 0<i\leq n \end{array}}x_{i}=B.$$

### Penalty Factors and Initial States

To incorporate the aforementioned constraint into the payoff equation, two different approaches have been considered for constructing the constraint in the QAOA module and the GAS module. Specifically, for the GAS module, the initial state and objective are defined as follows:

$$|\psi_{0}\rangle_{G}=\frac{1}{\sqrt{2^{n}}}\sum_{i=0}^{2^{n}-1}{|i\rangle }_{n},$$

$$F^{\left(0\right)}\left(x_{1},x_{2},\ldots ,x_{n}\right)=F\left(x_{1},x_{2},\ldots ,x_{n}\right)-P{\left(\sum_{\begin{array}{c} 0<i\leq n \end{array}}x_{i}-B\right)}^{2},$$

where the budget constraint is added as a penalty term to *F*, with *P* being the penalty coefficient.

The *P* should be chosen to be large enough s.t. $F^{\left(f\right)}>F_{max}^{\left(0\right)}>F^{\left(v\right)}$ for all infeasible states, yielding

$$F^{\left(v\right)}\left(x_{1},x_{2},\ldots ,x_{n}\right)=\max F\left(x_{1},x_{2},\ldots ,x_{n}\right),$$

$$F^{\left(f\right)}\left(x_{1},x_{2},\ldots ,x_{n}\right)=\max_{\sum_{\begin{array}{c} 0<i\leq n \end{array}}x_{i}=B}F\left(x_{1},x_{2},\ldots ,x_{n}\right).$$

In the credit card scoring problem, we choose $P\leq \frac{2^{m-1}}{{\left(\max\{n-B,B\}\right)}^{2}}$, s.t. the performance of GAS is not expected to deteriorate with the P. The *m* is the number of qubits available to store the result.

For the QAOA module, we set the Dicked state which satisfies the budget constraints as the initial state [8].

|ψ0〉M=1nB∑i1,i2,…,in∈{0,1}i1+i2+…+in=B|i1i2…in〉.

## 3. The Improved GAS Algorithm

**Figure 1:**
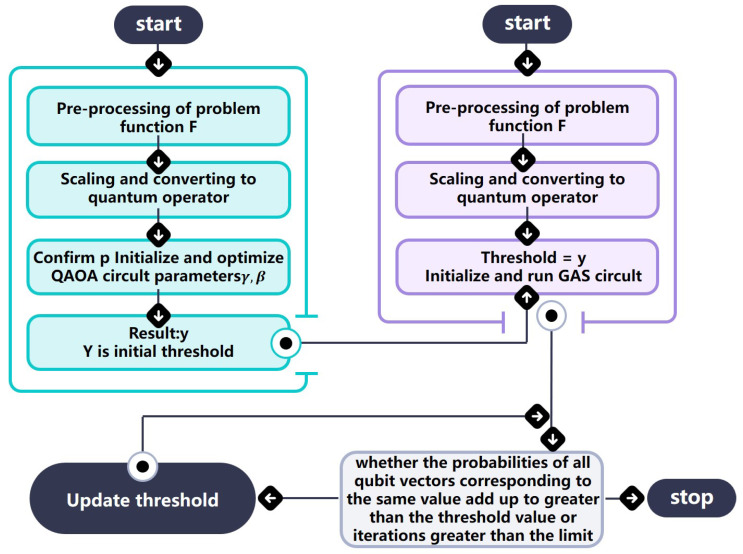
The chart shows all the steps of the improved GAS algorithm; the left part is the QAOA module which is used to calculate the initial threshold *y*. The right part is the GAS module which is used to receive and update *y* and calculate the final result.

The improved GAS algorithm combines the advantages of the QAOA and GAS algorithm to solve the CPBO problem. The overall flow of the algorithm is shown in Figure 1, our algorithm improves the original GAS with a better initial threshold selection.

### 3.1. The QAOA Module

QAOA can leverage quantum superposition to represent multiple solution states simultaneously in a single computational step, thereby improving search efficiency. Moreover, classical algorithms often rely on heuristic searching or gradient descent, making them prone to getting stuck in local optima. For NP-hard problems, solving them on classical computers requires exponential time, while QAOA theoretically offers the potential for exponential speedup. Finally, since this case involves a CPBO problem and we only need to obtain an approximate solution, QAOA not only theoretically demonstrates the possibility of polynomial speedup but also inherently possesses an approximate adiabatic evolution property, which makes it well suited to finding approximate solutions. Therefore, in our study, we used QAOA to construct the threshold value. To use the QAOA, we convert $F^{\left(f\right)}$ into a quantum operator.

(2) $${\hat{F}}_{QAOA}=F\left(\frac{\left({\hat{I}}_{1}-{\hat{Z}}_{1}\right)}{2},\frac{\left({\hat{I}}_{2}-{\hat{Z}}_{2}\right)}{2},\ldots ,\frac{\left({\hat{I}}_{n}-{\hat{Z}}_{n}\right)}{2}\right)=\sum_{\begin{array}{c} 0<i\leq n \end{array}}crt_{i}\left(1-h_{i}\right)\frac{\left({\hat{I}}_{i}-{\hat{Z}}_{i}\right)}{2}-ct_{i}h_{i}\frac{\left({\hat{I}}_{i}-{\hat{Z}}_{i}\right)}{2},$$

where ${\hat{Z}}_{i}$ denotes the Pauli-$\hat{Z}$ gate, acting on the *i*-th qubit. Due to the XY full mixer being able to ensure that the number of ones in the state remains unchanged, thus satisfying the constraint conditions, we use the XY full mixer to search for the optimal solution in all feasible states to explore a broader solution space and increase the probability of finding the global optimum. The XY full mixer is given as

$${\hat{U}}_{M}\left(\beta \right)=\prod_{\begin{array}{c} \left(i,j\right)\in S_{M} \end{array}}{\hat{R}}_{i,j}^{\left(XY\right)}\left(\beta \right),$$

and the ${\hat{R}}_{i,j}^{XY}$ are arranged into *n* subsets of $\frac{n-1}{2}$ commuting operations where i+jmodn=k in subset *k*. (For example, for *n* = 3, S = {(1, 3), (2, 3), (1, 2)} with the subsets {(1, 3)}, {(2, 3)}, {(1, 2)}.) If the *n* is even, we first generate the subsets for the n−1. The first step is to generate the subsets as described above. Then, for each subset, we add the missing pair of qubits. (For example, for the n=4, we add (2, 4) to the first subset {(1, 3)}, (1, 4) to the second, and so on. The resulting set is {(1, 3), (2, 4), (2, 3), (1, 4), (1, 2), (3, 4)}), where *M* represents the XY full mixer model. Under this model, the set *S* contains all pairs of qubits, where the order is chosen such that as many gates as possible can be performed in parallel, thus minimizing the depth of the circuit. If *n* is odd,

$${\hat{R}}_{i,j}^{\left(XY\right)}\left(\beta \right)=e^{i\beta ({\hat{X}}_{i}{\hat{X}}_{j}+{\hat{Y}}_{i}{\hat{Y}}_{j})}.$$

The QAOA algorithm uses adiabatic evolution to get the final result, with the following quantum state depending on the parameters $\vec{\gamma }=\left(\gamma_{1},\gamma_{2},\ldots ,\gamma_{p}\right)$ and $\vec{\beta }=\left(\beta_{1},\beta_{2},\ldots ,\beta_{p}\right)$, with *p* (*p* is the depth of the QAOA quantum circuit) being the number of iterations,

$$|\psi_{p}\left(\vec{\gamma },\vec{\beta }\right){>}_{M}={\hat{U}}_{M}\left(\beta_{p}\right)e^{-i\gamma_{p}\hat{F}}\ldots {\hat{U}}_{M}\left(\beta_{2}\right)e^{-i\gamma_{2}\hat{F}}{\hat{U}}_{M}\left(\beta_{1}\right)e^{-i\gamma_{1}\hat{F}}{|\psi_{0}\rangle }_{M}.$$

Finally, all qubits are measured with respect to the standard basis in order to determine the mean value,

$${\langle F\rangle }_{\vec{\gamma },\vec{\beta }}=\langle \psi_{\vec{\gamma },\vec{\beta }}|\hat{F}|\psi_{\vec{\gamma },\vec{\beta }}\rangle .$$

The mean value is then passed to a classical optimizer, which gives new values to the parameters γ and β to minimize the expectation ${\langle F\rangle }_{\vec{\gamma },\vec{\beta }}$.

**Figure 2:**
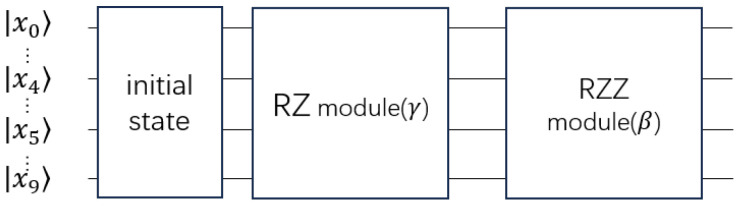
This circuit implies the equation ${\hat{U}}_{S_{M}}\left(\beta \right)e^{-i\gamma \hat{F}}$=$\prod_{\left(i,j\right)\in S_{M}}{\hat{R}}_{i,j}^{XY}\left(\beta \right)\prod_{\left(i,j\right)\in S_{M}}e^{-i\gamma W_{ij}{\hat{Z}}_{i}{\hat{Z}}_{j}}\prod_{i=1}^{n}e^{-i\gamma w_{i}{\hat{Z}}_{i}}$. The RZ module represents $\prod_{i=1}^{n}e^{-i\gamma w_{i}{\hat{Z}}_{i}}.$ The RZZ module represents $\prod_{\left(i,j\right)\in S_{M}}e^{-i\gamma W_{ij}{\hat{Z}}_{i}{\hat{Z}}_{j}}.$ $W_{ij}$ is the coefficient of the ${\hat{Z}}_{i}{\hat{Z}}_{j}$ in ${\hat{F}}_{QAOA}$; the $w_{i}$ is the coefficient of the ${\hat{Z}}_{i}$ in ${\hat{F}}_{QAOA}$.

The QAOA circult can be shown as Figure 2.

### 3.2. GAS Module

The GAS algorithm has three components:

1. The $A_{y}$ prepares an n-qubits input register to represent the equal superposition of all ${|x\rangle }_{n}$ and m-qubits output register to (approximately) represent the corresponding objective function ${|f\left(x\right)-y\rangle }_{m}$.
  $$A_{y}{|0\rangle }_{n}{|0\rangle }_{m}=\frac{1}{\sqrt{2^{n}}}\sum_{x=0}^{2^{n}-1}{|x\rangle }_{n}{|f\left(x\right)-y\rangle }_{m},$$
  where |x〉 is the binary encoding of the integer *x*.
2. The oracle *O* recognizes the states of interest and multiplies their amplitudes by −1.
  $${O|x\rangle }_{n}{|z\rangle }_{m}={sign\left(z\right)|x\rangle }_{n}{|z\rangle }_{m}.$$
3. The Grover diffusion operator *D* has the effect of flipping all amplitudes in the quantum state according to their mean. This causes all the amplitudes of the states of interest to be magnified, while the amplitudes of all other states are decreased. For more details on building the GAS algorithm, see the Appendix A.
  $$D=H^{⨂n+m+1}(2|0\rangle \langle 0|-I)H^{⨂n+m+1}.$$

Furthermore, applying the Grover operator $A_{y}DA_{y}^{†}O$*r* times to state $A_{y}{|0\rangle }_{n}$ for an integer r≥0 will maximally amplify the amplitudes of the states of interest.

To ensure the probability of sampling the target state of at least 1/2, the optimal number of *r* depends on the number of all states $M=2^{n}$ and the number of target states *N*, with $r=\lfloor \frac{\pi }{4}\sqrt{\frac{M}{N}}\rfloor .$

This is a quadratic speed-up with respect to the classical search. Since *N* is in general unknown, in this article, we take a randomised strategy to set *N*.

When we use the GAS algorithm to solve credit card scoring problems, it is necessary to convert the payoff equation into the matrix form.

(3) $$\begin{array}{cc} {\hat{F}}_{GAS} & =\sum_{\begin{array}{c} 0<i\leq n \end{array}}crt_{i}\left(1-h_{i}\right)x_{i}-ct_{i}h_{i}x_{i}-P{\left(\sum_{\begin{array}{c} 0<i\leq n \end{array}}x_{i}-B\right)}^{2}\\ & =\sum_{i,j=1}^{n}Q_{ij}x_{i}x_{j}+\sum_{i=1}^{n}b_{i}x_{i}+c.\;x_{i}=0,1, \end{array}$$

where $Q=\left[\begin{array}{cccc} -P & 0 & \cdots & 0\\ -2P & -P & \cdots & 0\\ \vdots & \cdots & \ddots & \vdots \\ -2P & -2P & \cdots & -P \end{array}\right]$,

$b=\left[\begin{array}{ccc} 2PB+crt_{1}\left(1-h_{1}\right)-ct_{1}h_{1} & \ldots & 2PB+crt_{n}\left(1-h_{n}\right)-ct_{n}h_{n} \end{array}\right].$

The improved GAS algorithm (Algorithm 1) pseudocode is given below:
**Algorithm 1:** Improved-GAS algorithm**Input:**  $f:k\leftarrow R^{+},\;\Theta_{0}=\left[0\ldots n\right],\;{\Theta_{0}\left[j\right]}_{j\in [0,n)}\in \left(0,1\right),\;\lambda >1$;**Output:** *y*1:set shot_QAOA=512;2:set $P=F_{v}$;3:set i=1;4:set shot_GAS=200 and max_iter=60;5:set threshold=0.7;6:**repeat**7:    Run QAOA circuit;8:    Measure result;9:    Update $\Theta_{0}$;10:**until** The difference between the updated $\Theta_{0}$ of the optimizer and the last $\Theta_{0}$ is less than a certain precision;11:12:Run QAOA circuit;13:$y=y_{1}=$ Measure result;14:15:**repeat**16:    Select the rotation count $r_{i}$ from the set {⌊k/2⌋,…,⌈k−1⌉} randomly;17:    Run the GAS quantum circuit and apply Grover Search with $r_{i}$ iterations using oracles $A_{y_{i}}$ and $O_{y_{i}}$. We denote the outputs *y* respectively;18:    **if** $y<y_{i}$ **then**19:        y=$y_{i}$;20:        ans = $y_{i}$;21:    **else**22:        ans=y;23:        i=i+1;24:    **end if**25:**until** The i=max_iter or *y* correspond to all of the frequencies greater than the threshold;

## 4. Test Case

In this section, we present an experiment. Currently, the credit card scoring problem we considered includes 1000 credit card data, and the existing machines are not enough to run that amount of data at the same time. So we have to divide the credit card data into 200 groups and test with 10 cards’ data each time. All of the following experiments are performed with the initial y=10 in the GAS module and p=2 in the QAOA module.

**Figure 3:**
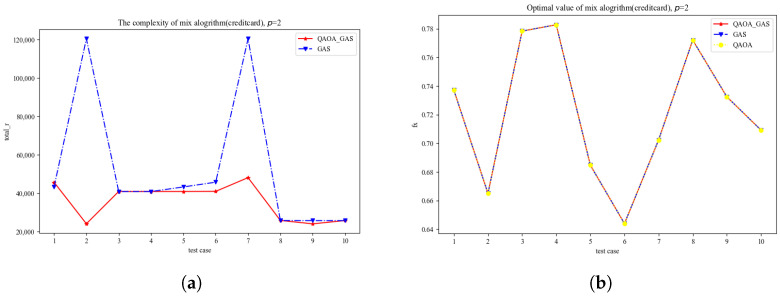
(**a**) is speed chart of the credit card scoring problem; (**b**) is accuracy chart of credit card scoring problem.

Figure 3a,b represent the result of the credit card scoring problem. In Figure 3a, the blue line represents the time complexity of the GAS algorithm, which is calculated by the actual number of iterations multiplied by the quantum circuit depth, and the red line represents the time complexity of the improved GAS algorithm, which is obtained by adding the complexity of the GAS algorithm to the complexity of the QAOA algorithm; the time complexity of the QAOA is obtained by multiplying the number of layers *p* by the maximum number of updates of Θ. The Y-label represents the total complexity, and the X-label represents the different test cases. According to Figure 3a, we can conclude that the time complexity of the improved GAS algorithm is better than that of the GAS algorithm. In Figure 3a, there are three different-colored lines, each line represent the improved GAS algorithm, the GAS algorithm and the QAOA. The Y-label represents the value of the algorithm, the X-label still represents the different test cases. It can be seen from the figure that the accuracy of the improved GAS algorithm is still consistent with the other two algorithms.

**Figure 4:**
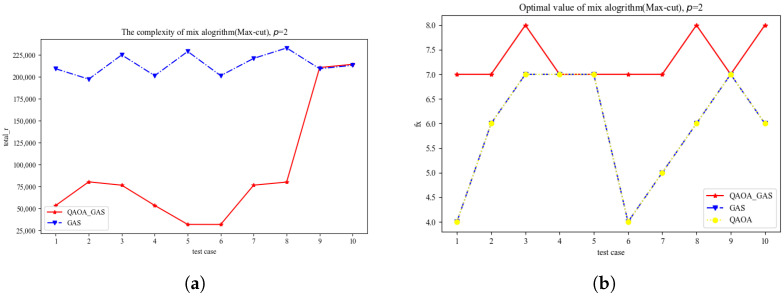
(**a**) is speed chart of the Max-Cut; (**b**) is accuracy chart of the Max-Cut.

Next, we test our algorithm on a Max-Cut problem with 20 nodes and 12 edges, as shown in Figure 4a,b. Obviously, in the QUBO problem, the improved GAS algorithm performs better than the GAS algorithm in time complexity. Similarly, the improved GAS algorithm has advantages over the QAOA algorithm in terms of accuracy. Note that the accuracy of the improved GAS algorithm should theoretically be the same as that of the GAS algorithm. However, in this experiment, we set the max-iteration which causes both the GAS algorithm and the improved GAS algorithm to stop before turning over the correct result. For this reason, the GAS algorithm or the enhanced GAS algorithm may not achieve the desired accuracy. Overall, the experiment achieved the expected results. The algorithm used a shallow QAOA algorithm to find the solution, which was faster than both the deep QAOA algorithm and non-quantum algorithms. Compared with the GAS algorithm, its time consumption was negligible.

Moreover, unlike the GAS algorithm, where the threshold value is either randomly chosen or manually set, the new algorithm avoids this issue. The GAS algorithm determines whether a condition is met by checking the sign bit. If the initial threshold is lower than the solution value, the iteration will continue indefinitely unless a maximum iteration count is set. Conversely, if the threshold is higher than the solution value but the gap is too large, the iterative update logic may lead to excessive iterations.

As previously discussed, the time required for a single iteration of the entire algorithm model is approximately $\left\lfloor \frac{\pi }{4}\sqrt{\frac{M}{N}}\right\rfloor$. The formula indicates that if a good initial value is provided, the time complexity increases as the number of iterations grows. This explains why the execution time of the new algorithm has been significantly reduced.

## 5. Conclusions

In this paper, we introduce an improved GAS algorithm which uses QAOA to generate the initial threshold *y* applied to the GAS algorithm to solve the CPBO problem. Our approach improves the speed of the GAS algorithm without affecting the accuracy. In this study, we demonstrate the efficacy of our algorithm in two distinct domains: the credit card scoring problem and Max-Cut problem. In this manuscript, we focus on integers and two decimal places since, as the number of decimal places increases, the number of qubits required must also increase, potentially exceeding the computing power of existing machines. Since this algorithm can be adapted to credit card problems, and credit card data typically consist of both an integer part and a decimal part (rounded to two decimal places), the model can be fully applied to search problems with similar data characteristics. Examples include stock selection problems, real bounding box filtering in object detection, and more.

Furthermore, by improving the quantum circuit construction of the algorithm and leveraging advancements in quantum hardware, it will be possible to process data with more decimal places. In the NISQ era, this search algorithm provides a more efficient approach to handling CPBO problems and offers a new solution for data cleaning and classification in the AI era.

This algorithm can serve as a valuable complement to existing methods and play a significant role in CPBO problems.

## Figures and Tables

**Table 1:** Notations.

Notation	Description
$F(x_{1},x_{2},\ldots ,x_{n})$	Payoff function
n	The number of credit card
c	Total loan of each card
r	Credit card interest rate
$t_{i}$	Card i loan approval rate
$h_{i}$	Bad debt rate for card i

**Figure A1:**
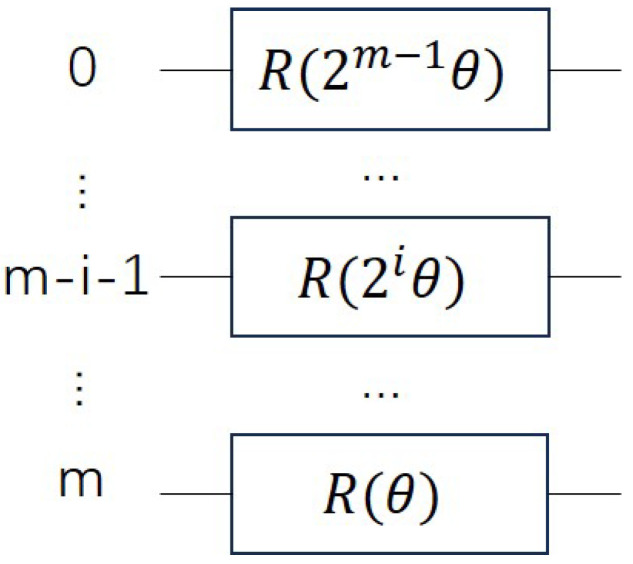
A circuit for unitary operator $U_{G}\left(\theta \right)$, where θ∈[−π,π) is applied to an m-qubit register. The symbol *R* denotes the phase gate, which rotates the amplitudes of states containing 1 in the position corresponding to the qubit to which it is applied.

**Figure A2:**
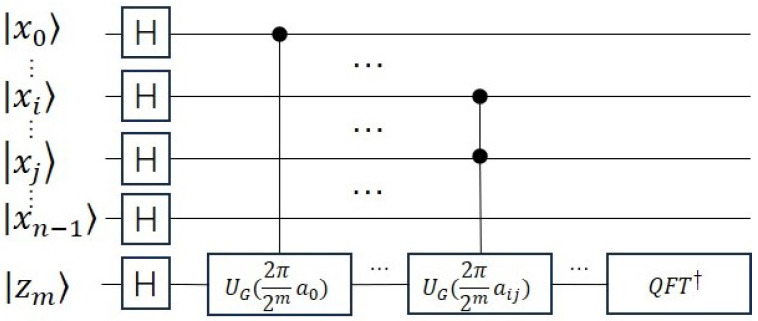
This circuit represents operator *A*, each $U_{G}$ operator represents the non-zero term in ${\hat{F}}_{GAS}$, and the main function of the circuit is the encode of an integer $k\in [-2^{m-1,2^{m-1}})$, which can apply to a register of m-qubits in an equal superposition.

**Figure A3:**
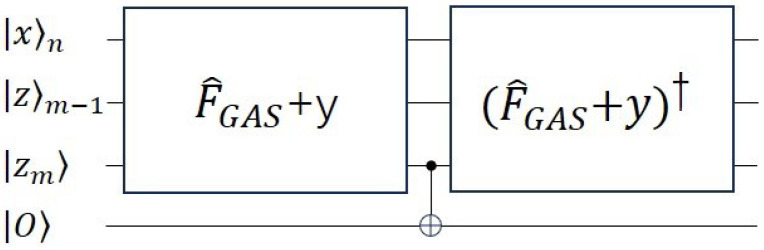
In this circuit (the ${\hat{F}}_{GAS}+y$ module contains operator *D*), there are n binary variables in the ${\hat{F}}_{GAS}+y$, and the global indicator qubit (shown at the bottom of the circuit) is set to |1〉 if and only if ${\hat{F}}_{GAS}+y$—where *y* is the threshold parameter from the GAS algorithm that is less than 0.

## Data Availability

The data presented in this study are available on request from the corresponding author.

## Appendix A

### Appendix A.1

The main idea of the GAS algorithm is to construct Ay and Oy for a given threshold *y* such that the *y* flag all states x ∈ {0,1} satisfying f(x)<y, such that we can use the Grover algorithm to find a solution $\hat{x}$ that makes $f(\hat{x})$ smaller than *y*. Then, we set the $y=f(\hat{x})$ and repeat the process until some formal termination criteria are met.

### Appendix A.2. Construct Operator A

In this section, we show how to construct the operator *O*. Due to the m-register representing the corresponding $|{\hat{F}}_{GAS}{-y\rangle }_{m}$, we have to use a relatively simple structure to achieve this operator. The simplest implementation is to use the phase gate R(θ): when the gate is applied to one qubit, it rotates the phase of the amplitudes of the states, having 1 in the position corresponding to that qubit.

Consider that if there is a $kx_{i}x_{j}$ term that exists in ${\hat{F}}_{GAS}$, when we use matrix restore *k*, the *k* can be expressed as $a_{ij}$. If we apply $UG(\frac{2\pi }{2^{m}}k)$ followed by the inverse Quantum Fourier Transform (QFT) to an m-qubit register, we end up with $k(mod2^{m})$ being encoded in the register.

Note that credit card scoring problems have a degree of monomials less than or equal to 2, so we only need to control single qubits and pairs of qubits (i.e there only exists $a_{i}$ or $a_{ij},i,j\in \left[0,n\right)$ in ${\hat{F}}_{GAS}$).

### Appendix A.3. Construct Operator O

At each stage of the algorithm, a fixed coefficient is incorporated into the polynomial, and the remaining negative values are identified. This implies that the oracle is only required to recognize negative integers. Since values are represented in complement-on-two, where the most significant (left-most) bit designates the sign of the number, a single quantum bit in the value register can be employed to identify negative integers. The conventional oracle that multiplies target amplitudes by −1 may be utilized. It should be noted that the oracle remains unchanged throughout the iterations, as the addition of a constant to the polynomial may result in overflow within the value register. Consequently, in order to circumvent this issue, it may be necessary to increase the number of qubits in the value register by one.

### Appendix A.4. Construct Operator D and Update y

The operator *D* is the last stage of algorithm. When we combine all the operations together, we get the ${\hat{F}}_{GAS}+y$ from the quantum circuit. The circuit of Figure A3 is executed in order to achieve the desired counts. In the event that the counts are divided by the total number of samples, and the result is less than the set threshold, the circuit must be repeated until the algorithm reaches the maximum number of iterations, or the threshold is exceeded. It is possible for it to be observable that multiple maxima have the same value. In order to address this issue, a pruning operation has been implemented which involves determining whether the same frequency corresponds to the same value and then selecting one of them then terminate the algorithm.
